# Comparative assessment of green and chemically synthesized glutathione capped silver nanoparticles for antioxidant, mosquito larvicidal and eco-toxicological activities

**DOI:** 10.1038/s41598-023-35249-7

**Published:** 2023-05-19

**Authors:** Radha Yadav, Shabad Preet

**Affiliations:** Department of Zoology, Faculty of Science, Dayalbagh Educational Institute, (Deemed to be University) Dayalbagh, Agra, 282005 India

**Keywords:** Zoology, Diseases, Health care

## Abstract

A comparative assessment of AgNPs synthesized through three different routes viz*.* clove bud extract mediated AgNPs, sodium borohydride AgNPs and Glutathione (GSH) capped AgNPs for antioxidant and mosquito larvicidal activities was the major focus of the present study. The nanoparticles were characterized using UV–VIS spectrophotometry, dynamic light scattering (DLS), X-ray diffraction (XRD), field emission-scanning electron microscopy (FE-SEM), transmission electron microscopy (TEM) and Fourier Transform Infrared Spectroscopy (FTIR) analysis. Characterization studies revealed the synthesis of stable, crystalline AgNPs measuring 28 nm, 7 nm and 36 nm for green, chemical and GSH-capped AgNPs respectively. FTIR analysis exhibited the surface functional moieties that were responsible for reduction, capping and stabilizing AgNPs. Antioxidant activity was found to be 74.11%, 46.62% and 58.78% for clove, borohydride and GSH-capped AgNPs respectively. Mosquito larvicidal bioactivity of AgNPs against *Aedes aegypti* IIIrd instar larvae depicted clove AgNPs being most effective (LC_50_—4.9 ppm, LC_90_—30.2 ppm) followed by GSH-capped (LC_50_—20.13 ppm, LC_90_—46.63 ppm) and borohydride AgNPs (LC_50_—13.43 ppm, LC_90_—160.19 ppm) after 24 h. Toxicity screening against aquatic model *Daphnia magna* revealed Clove mediated and GSH-capped AgNPs to be safer as compared to the borohydride AgNPs. It may be envisaged that green and capped AgNPs may be further explored for diverse biomedical and therapeutic applications.

## Introduction

Nanotechnology allows the manipulation and modification in the properties of particles when they are synthesized implementing different routes. Applications of nanoparticles are tremendous and are increasing every year in multiple folds. We need to utilize this technology wisely in such a way that it poses no threat to the environment and organisms. Even with such huge advancements and progress in the field of medicine, science and research, mosquito borne diseases are still affecting millions of populations warranting an urgent attention to control this warfare of mosquitoes. Since, no effective vaccine or drug is available for controlling this disease, the solution of the problem lies in effective management of vector.

For instance, Dengue is an arthropod borne viral disease, spread mostly by the bite of infected *Aedes aegypti* and upto a lesser extent by *Aedes albopictus* bite as well. The global incidence of dengue has grown dramatically in the last few years, putting about half of the world's population at risk. As per the recent report by WHO, around 350–400 million dengue infections occur each year, of which 96 million (67–136 million range) manifest clinically^1^. Apart from active cases, it is estimated that 3.9 billion people are at risk of infection with dengue viruses. Despite a risk of infection existing in 129 countries, 70% of the actual burden is in Asia^2^. Before 1970, only 9 countries had experienced severe dengue epidemics^3,4^. The disease is now endemic in more than 100 countries. Also, the co-infection of dengue and COVID-19 have devastating consequences on the populations at risk, though this field requires more insights into the biology of both diseases, as there are substantial challenges due to overlapping clinical and laboratory parameters for both the diseases^5^.

For this, the most promising nanotechnology is gaining popularity through the use of nano-sized materials for controlling excessively proliferating mosquito populations. It deals with controlling the matter at molecular scale in the range of 1 to 100 nm and its immense applications have captured the mosquito management field. Prominent use of nanoparticles is because of their novel physico-chemical and biological potentials. Nanoparticles are prepared using metals like gold, silver, copper, zinc, cadmium and various others metals (iron, magnesium, platinum, titatnium etc.). Silver metal has gained popularity in nanoparticle synthesis due to their unique physical and chemical properties. Apart from physico-chemical properties, AgNPs also have important biological properties like, antioxidant, antibacterial, antifungal, antiviral, anti-inflammatory, anti-cancer, anti-angiogenic and anthelminthic^6,7^. Due to potential applications of AgNPs in mosquito control, researchers have discovered various ways to synthesize them via physical, chemical, photochemical and various other methods. In spite of having tremendous larvicidal activity of AgNPs synthesized via., physico-chemical routes, they have problem of cluster formation, aggregation, large size and instability^8^. They are also associated with a major and growing concerns of toxicity for the environment. Although reduction of particle size from micro to nano offers more unique physical properties but also makes the particle more toxic. They are known to cause toxicity in aquatic organisms and may induce mutations, cancer and even death in non-target organisms viz*.,* fishes, other aquatic organisms including their embryos. NPs enter into the food chains and trophic level and become incorporated into food chains and therefore into human bodies causing various health problems, as they are not stabilized and cause generation of free radicals in body^9^. As this is a rapidly evolving technology, use of such materials will continue to mature, so will the problem of toxicity, therefore development of eco-friendly nanomaterials is the need of the hour which should be taken into consideration right from their synthesis. Hence, there are several aquatic animal models such as *Daphnia magna,* a fresh water filter-feeding crustacean. Since, in freshwater aquatic ecosystem food chain, it plays an important role of primary consumer, it is considered as a best model organism for eco-toxicity studies. Any form of alteration in their population is a good indicator of aquatic toxicity^10^.

Consequently, stabilization of nanoparticles could be the solution to the problem and this can be achieved by coating or capping AgNPs to cover their unstabilized surface. There are several capping agents viz*.* ligands, surfactants, biomolecules, thiols, polymers, dendrimers, etc. Thiol capping has a strong affinity for AgNPs and thiolated antioxidants such as l-cysteine and Glutathione (GSH) provide major physiological defence against the oxidative stress and damage^11^. Thiols are found to have a strong affinity for the silver, so they tightly get bind to AgNPs causing their stabilization^12^. Besides, they also possess high radical scavenging efficacy hence, can act as antioxidants. Therefore, the free radical generation problem of nanoparticles can be tackled by stabilizing them with the help of capping agents. The interactions of AgNPs with sulphur atoms of GSH are very strong to allow immobilization of thiolated species and make them act as radical scavengers and thus provide major physiological defence against oxidative damage occurring in body.

Another approach of developing safer AgNPs is the green method. Utilizing the untapped potential of plants to prepare AgNPs is economical as well as safer, as they do not have high energy demand (temperature and pressure) and involvement of toxic chemicals in their preparation. In the green AgNPs synthesis, various compounds found in plant part extract acts as reducing agent and capping agents. Till date various researchers across the world have attempted the synthesis of AgNPs through bio-reduction process following green approach. Recently, several non-medicinal and medicinal plants have been used for green synthesis of AgNPs^13,14^.

Clove (*Syzygium aromaticum*) (L.) is a well-known spice, belonging to the Myrtaceae family. It is rich in many phytochemicals; sesquiterpenes, monoterpenes, hydrocarbon, and phenolic compounds. Eugenyl acetate, eugenol, and β-caryophyllene are the most significant phytochemicals found in clove, which are responsible for its great range of biological activities. This plant is rich in thiol moieties due to eugenol (4-allyl-2-methoxyphenol) which acts as reducing and capping agent. Clove extract and oil has been tested for their analgesic, antioxidant, anticancer, antiseptic, anti-depressant, antispasmodic, anti-inflammatory, antiviral, antifungal, and antibacterial activity^15^. Very few studies have been reported for the mosquito larvicidal potential of clove oil, and none on clove nanoparticles, to the best of our knowledge. Therefore, to fill this gap, clove was used for the green route of NPs synthesis in the present study.

Hence, in the present work, three different types of nanoparticles, viz*.* clove mediated green AgNPs, borohydride AgNPs and a third type of surface stabilized; GSH-capped AgNPs were synthesized and the effect of capping on morphology, stability of AgNPs was studied using various characterization techniques along with their comparative evaluation of antioxidant and dengue vector larvicidal activities. The eco-toxicological study of these AgNPs was also evaluated on a fresh water crustacean *Daphnia magna* for validating level of associated risk.

## Results and discussion

### Synthesis and characterization of AgNPs

This study was aimed for the development of stable, feasible and eco-friendly AgNPs, with good bioactivity. This was done in two ways—preparing AgNPs using clove, as a green approach, using plant material and GSH capping for surface modification of chemically synthesized AgNPs for making them effective, safe and stable. Synthesis of clove AgNPs was visualized through a distinct change in colour of AgNO_3_ solution from colourless to dark brown with the addition of aqueous clove extract. Eugenol (4-allyl-2-methoxyphenol), which is the main compound found in clove extract, is responsible for the reduction of silver nitrate and formation of clove AgNPs. A proton is released from OH group of eugenol, creating negative charge on it. The release of two electrons from resonating structure of anionic form of eugenol in this process are responsible for the reduction of 2 Ag+ ions to 2 Ag0. Eugenol, also acts as a capping agent and stabilizes the formed AgNPs^16^.

For the nanoparticles prepared using sodium borohydride, colour change from transparent to yellow indicated the formation of AgNPs. Herein, sodium borohydride facilitated the reduction of AgNO_3_ solution. While for the synthesis of GSH-capped AgNPs, dark yellow colour after addition of capping agent-GSH, indicated the completion of capping process. The surface of AgNPs is positively charged, while the acetyl (–COO) and thiol (–SH) group of GSH are negatively charged. So, the stabilization of [Ag(GSH)] is due to formation of van der walls forces between these opposite charges. Ionic surface stabilizing agent adsorbs on the surface of AgNPs and generates uniform charge on particle surface, this results in the formation of uniform and stable size particles, with no aggregation^17^.

### UV–Vis spectrophotometric analysis

**Figure 1:**
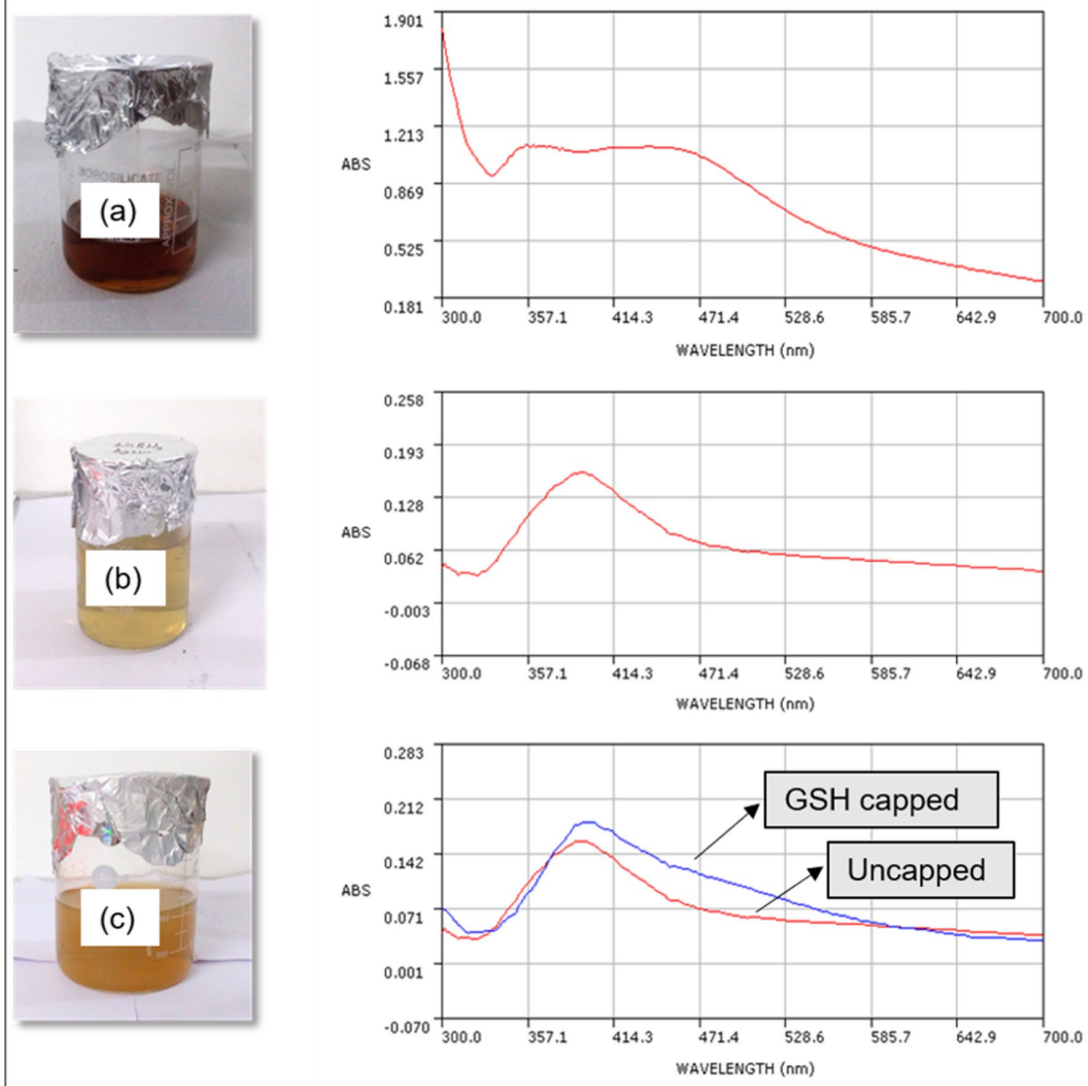
Synthesized AgNPs and their respective UV–Vis absorption spectra (**a**) Clove AgNPs, (**b**) Borohydride AgNPs, (**c**) GSH-capped AgNPs and borohydride (uncapped) AgNPs.

Synthesis of AgNPs was monitored using UV–Vis Spectral analysis. Spectral peaks of synthesized AgNPs are shown in Fig. 1. Spectral peak of green synthesized AgNPs using clove extract was recorded at 439.2, while borohydride AgNPs showed a distinct peak at 388 nm. The spectral peak of GSH-capped AgNPs was observed at 400.8 nm. Our spectral study for GSH- capped is in line with the earlier reported work^18^. AgNPs exhibit a characteristic surface plasmon resonance (SPR) band at or around 400 nm. Spectral peaks of all three types of AgNPs in our study were near or around 400 nm, confirming the formation of AgNPs.

### DLS and zeta potential analysis

**Figure 2:**
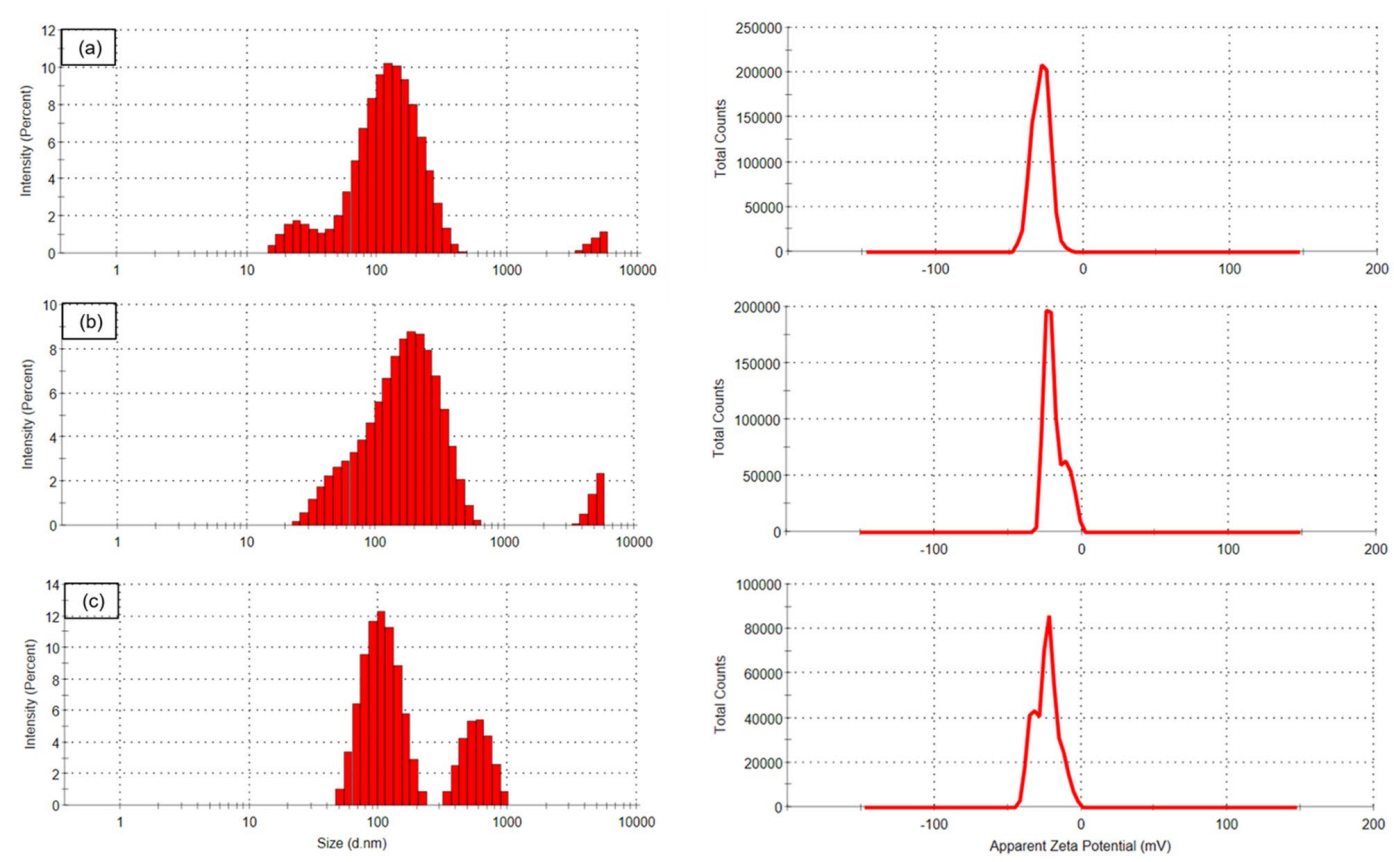
DLS- Particle size distribution and Zeta potential of (**a**) Clove AgNPs, (**b**) Borohydride AgNPs, (**c**) GSH-capped AgNPs.

DLS-size distribution and zeta potential studies were done to examine the hydrodynamic size, type of charge and stability of synthesized AgNPs, as shown in Fig. 2. Average hydrodynamic size for clove AgNPs was 100.0 nm, while for chemically synthesized borohydride AgNPs, the size was found to be 136.4 nm. GSH-capped AgNPs size was found to be 173.3 nm, which was larger as compared to the size of borohydride AgNPs. The pdI values of clove, borohydride and GSH-capped AgNPs were measured as 0.36, 0.52 and 0.41 respectively indicating their polydisperse nature.

Stability of all three types of synthesized AgNPs was examined through zeta potential. For AgNPs to be stable the value of zeta potential should be around ± 30 mV. The values obtained for clove AgNPs, borohydride AgNPs and GSH-capped AgNPs were as follows: − 28.5 mV, − 18.6 mV and − 23.5 mV. All the values were negatively charged and sharp, showing the surface of AgNPs to be negatively charged and dispersed in medium. The negative value also shows high electrostatic repulsion among the nanoparticles, thus preventing agglomeration and making them highly stable. All three types of AgNPs exhibited high zeta potential, however, clove AgNPs were found to be most stable, with highest zeta potential value. Moreover, zeta potential value of GSH-capped AgNPs was higher compared to borohydride AgNPs clearly indicating the importance of capping in adding stability to AgNPs.

### XRD analysis

**Figure 3:**
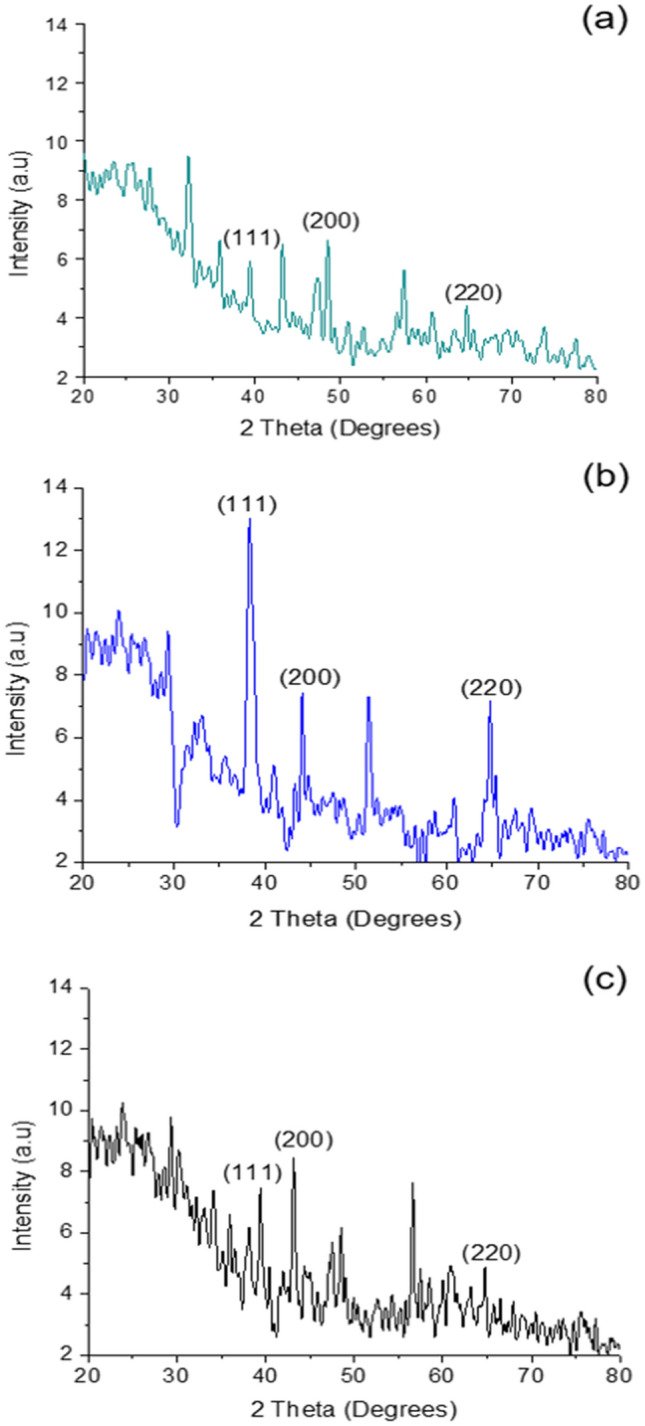
XRD diffraction spectra of (**a**) Clove AgNPs, (**b**) Borohydride AgNPs, (**c**) GSH-capped AgNPs.

Figure 3 shows the XRD data, which confirms the crystalline nature of the synthesized AgNPs. XRD data shows the diffraction peaks at 38.09°, 44.59° and 64° corresponding to (111), (200) and (220) planes of face-centred cubic crystal structure of metallic silver. This was found with all three types of synthesized AgNPs.

### FE-SEM and EDX analysis

**Figure 4:**
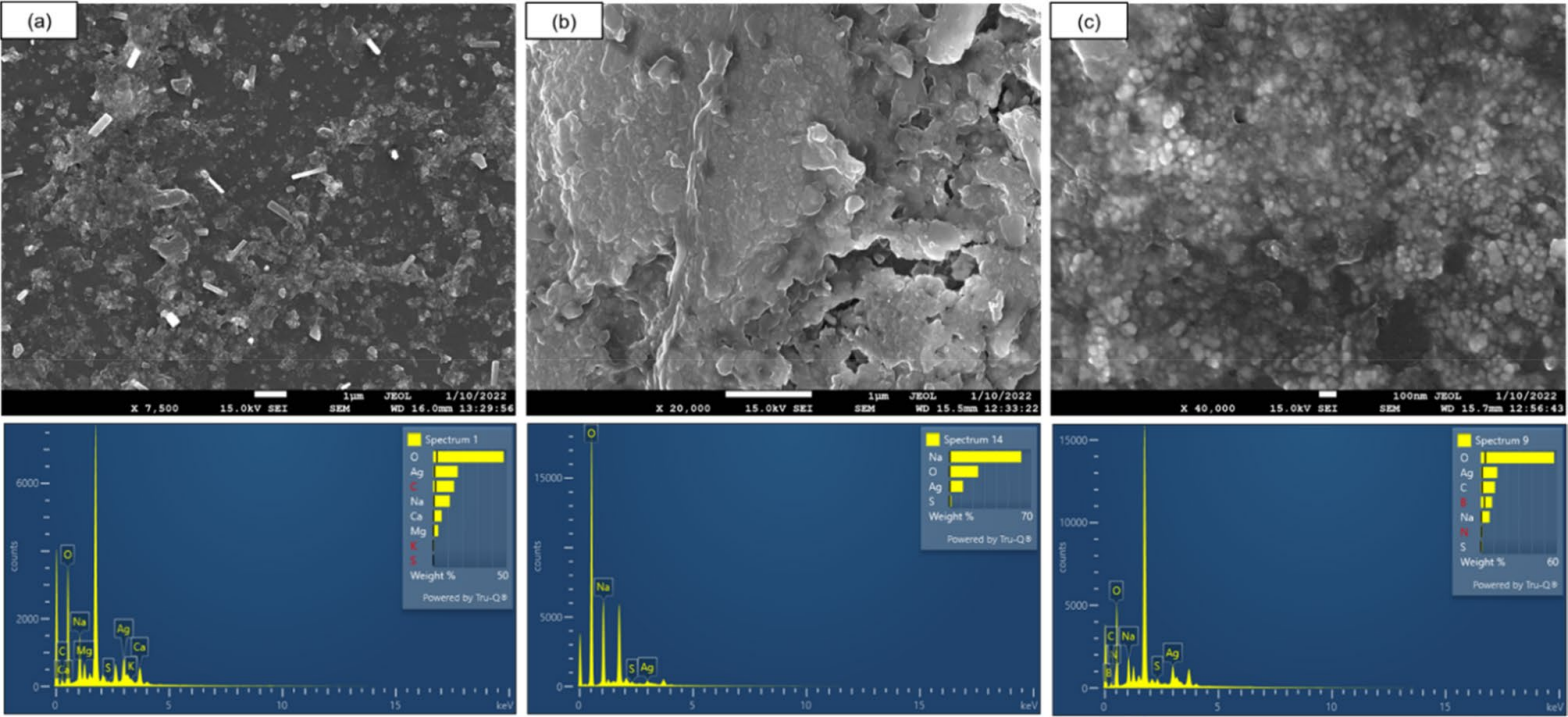
FE-SEM and EDS-spectra confirming presence of silver in AgNPs of (**a**) Clove AgNPs, (**b**) Borohydride AgNPs, (**c**) GSH-capped AgNPs.

FE-SEM clearly shows the shape and morphology of synthesized AgNPs, as shown in Fig. 4. The clove AgNPs were roughly spherical and evenly distributed. Few rod-shaped individual particles were also seen in clove AgNPs, but majority of particles were spherical in shape (Fig. 4a). Borohydride AgNPs were roughly spherical and aggregated (Fig. 4b). While chemically capped AgNPs were spherical, evenly distributed with small size (Fig. 4c). EDX analysis revealed presence of silver in all three types of synthesized AgNPs, Ag element shows a characteristic signal peak at 3 keV, due to surface plasmon resonance, which indicates the presence of silver in the system^19^. Silver (Ag) representative signal peak was observed near 3 keV (Lα) in all the AgNPs under study. Figure 4 also shows the quantitative element details of different AgNPs. The presence of elements apart from main Ag element like Na, Ca and Mg in clove AgNPs could be accounted to secondary metabolites present in clove extract, which are very crucial for nanobiosynthesis of AgNPs. Similar results have been reported for AgNPs synthesized using *Pedalium murex* leaf, which also had presence of elements like O, C, K, Ca, Cl and Na. This may be accounted for metabolites and functional moieties present in *Pedalium murex* leaf extract used for preparing AgNPs^20^. Apart from this presence of C and O element in borohydride and GSH-capped AgNPs could be accounted to the grid used in FE-SEM study.

### TEM analysis

**Figure 5:**
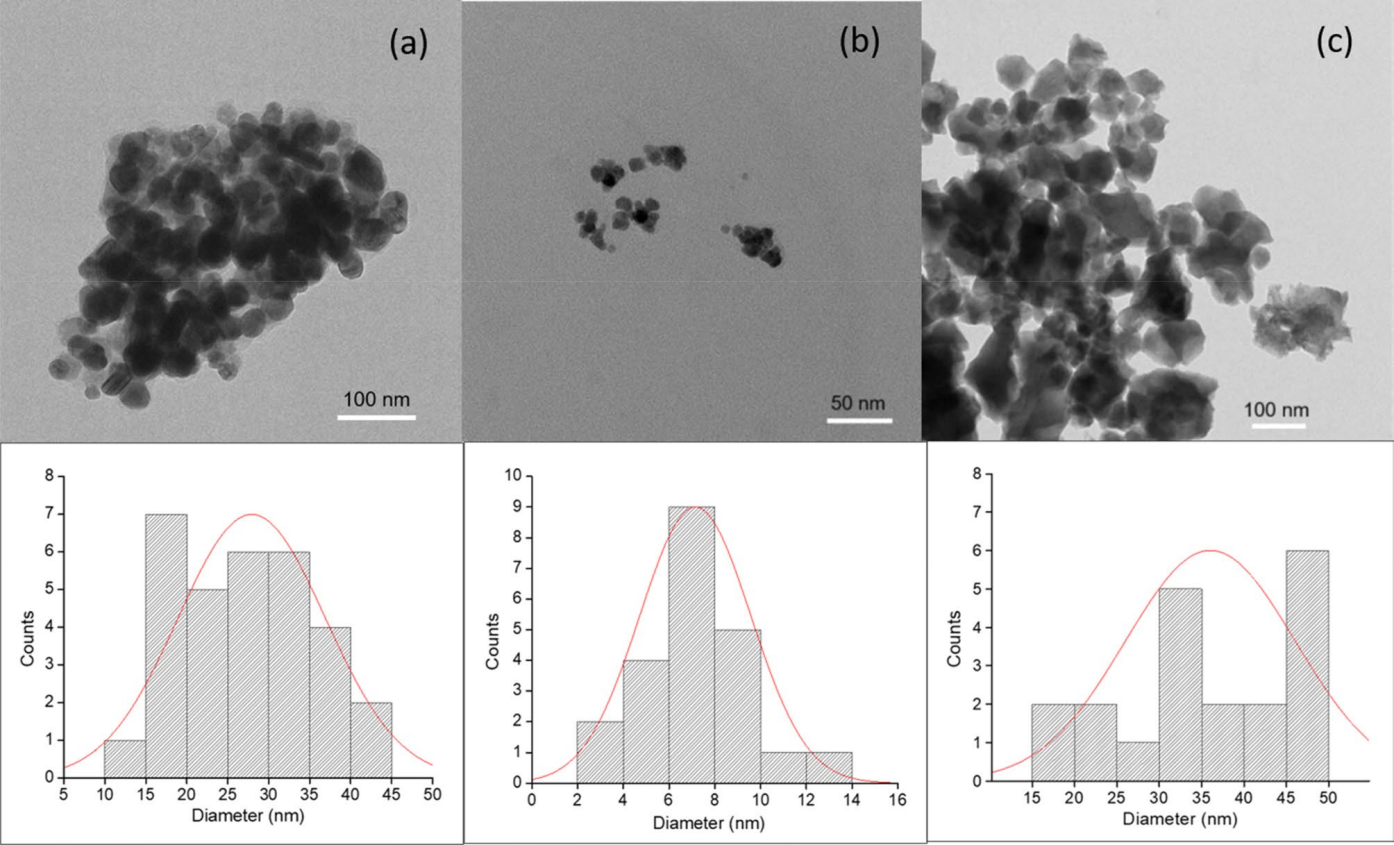
Transmission electron micrographs and size distribution histograms of (**a**) Clove AgNPs, (**b**) Borohydride AgNPs, (**c**) GSH-capped AgNPs.

TEM was done to study in details, the size and distribution of particles. TEM micrographs and the corresponding particle size distribution histogram of AgNPs obtained by TEM images are presented in Fig. 5. TEM micrographs of clove AgNPs clearly showed spherical shape AgNPs, with size around 28 nm (Fig. 5a). Similar size range was also found in AgNPs prepared using extracts of *Tectona grandis* seeds extract^21^. Possible reasons for the similar size range could be accounted to similar concentrations of extract used in preparation of AgNPs and presence of various phytopotentials moieties in the prepared AgNPs, due to the use of green compounds. Chemically synthesized AgNPs were spherical measuring 7 nm (Fig. 5b) whereas, GSH-capped AgNPs revealed spherical particles with slight aggregation measuring around 36 nm (Fig. 5c). The comparison between the sizes of borohydride AgNPs and GSH-capped AgNPs, clearly shows the role of GSH capping in enhancing the particle size.

Results of particle size obtained from DLS studies were larger compared to their size revealed through TEM results. Our results found correlation with study on AgNPs prepared using *Pedalium murex* leaf extract, in which DLS size was 23 nm larger than the TEM size of prepared AgNPs^20^. In another case, of Zirconia nanoparticles, the difference in size of nanoparticles given by DLS was higher by a factor of two to three compared to size analyzed for same nanoparticles by TEM technique^22^. Larger size found in DLS studies compared to TEM could be explained with the fact that TEM analysis delineates actual particle size while DLS provides hydrodynamic size representing a sum of particles, hydration sphere and surfactant agent shell coverage hence, relatively larger size of nanoparticles. Moreover, DLS is measured in solution form, with a solvent layer, and thus has tendency to aggregate, while for TEM analysis samples are dried, and thus provides actual size.

### FTIR analysis

**Figure 6:**
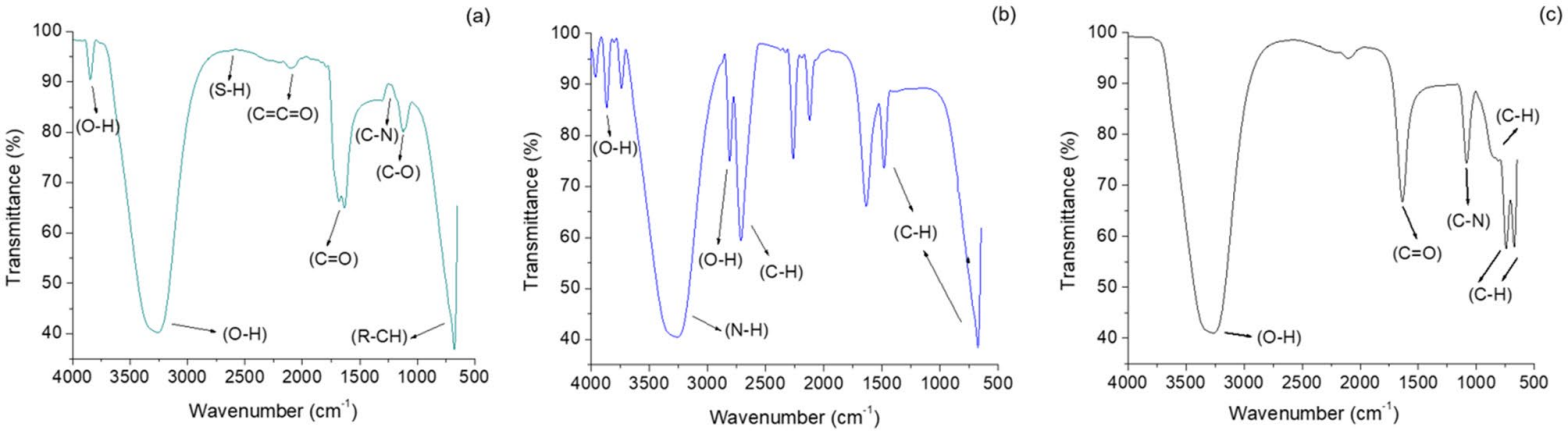
FTIR spectra of (**a**) Clove AgNPs, (**b**) Borohydride AgNPs, (**c**) GSH-capped AgNPs.

Figure 6 presents FTIR analysis which provides information about different functional groups and this spectral study is important for deciphering interactions resulting in reduction, adsorption and formation of AgNPs. Analysis was done in the spectral window of 500 to 4000 cm^−1^. FTIR spectra of clove AgNPs was done to elucidate knowledge about the chemical changes occurring in functional moieties of phytopotentials of clove bud extract, upon being adsorbed on the AgNPs surface and their participation in bioreduction of silver metal. A small peak was recorded at nearly 3700 cm^−1^ corresponding to O–H stretching of free hydroxyl groups of alcohol and phenols. Apart from this, a broad peak was also observed between 3550 and 3200 cm^−1^ corresponding to intermolecular bonded O–H group of residual eugenol^16^. A small peak was observed at 2600 cm^−1^ corresponding to S–H thiol group. Also, a tiny stretch found at 2150 cm^−1^ which may be accounted for ketene group. From literature, it is evident that band at 1650 cm^−1^ corresponds to C=O is not found in clove extract, while appearance of this band in clove AgNPs spectra is proof of involvement of eugenol in bioreduction process and formation of AgNPs^23^. Thus, it indicates the formation of clove AgNPs capped with different bio moieties^24^. The IR spectrum showed a peak at 1098 cm^−1^ corresponding to C–O (carboxylic acid derivative) stretching, formed in eugenol after bioreduction of silver. A very sharp and intense peak was obtained at 650 cm^−1^ corresponding to R-CH alkane functional group, which are abundantly found in clove extract. These observations confirmed the presence of eugenol and other functional moieties (mainly water-soluble flavonoids), which acts as reducing and stabilizing agent in the synthesis of clove AgNPs.

In borohydride AgNPs, several peaks with high intensity were obtained because of interaction of chemical NaBH_4_ with AgNO_3_. Stretches of O–H were also recorded between 3700 and 3500 cm^−1^ corresponding to free hydroxyl and H-bonded alcohol and phenol groups. A broad stretch was recorded between 3500 and 3300 cm^−1^ corresponding to N–H aliphatic primary amine. Peaks recorded at 2800 cm^−1^ corresponded to aliphatic C–H bending vibrations while a small, weak band was observed at 2550 cm^−1^ upper side, corresponding to S–H thiol group. A Significant peak was detected at 2260 cm^−1^, which could be attributed to C≡N nitrile stretching whereas, a small vibration was observed at 2100 cm^−1^, corresponding to alkyne (C≡C) however, a sharp and strong peak was noticed at 1620 cm^−1^, which could be assigned to C=C α, β unsaturated ketone. A very sharp and intense peak was also obtained at 1423 cm^−1^ which could be accounted to be generated by the borohydride anion of NaBH_4_, that remained partly bound to the AgNPs and corresponded to C–H bending. Moreover, stretch at 800 cm^−1^ corresponds to aliphatic C-H bending vibrations of alkanes.

The interactions of AgNPs with GSH were confirmed by the absence of N–H and S–H stretching bands, which are otherwise present in pure GSH and found at 3350 cm^−1^ and 2600 cm^−1^ respectively^11^. The absence of spectral peaks of S–H and N–H, suggest that glutathione is modified onto the surface of AgNPs by the thiol and amine groups from the cysteine moiety of Glutathione. These findings are supported by a previous study where a small spectral stretch resembled our FTIR analysis^17^ however, as different conditions such as heating and longer stirring hours were used by them, there were some deviations in the present spectra. Additionally in the study, a broad peak was observed between 3550 and 3200 cm^−1^ corresponding to O–H stretching of intermolecular bonded alcohol. Also, a small stretch at 2140 cm^−1^ was found, which can be assigned to alkyne (C≡C). Apart from this, a very sharp and intense peak was obtained at 1650 cm^−1^ for C=O stretch representing α, β unsaturated aldehydes and ketones. One more distinct peak was also obtained at 1250 cm^−1^ which represents C–N functional group of aliphatic amines. Vibration of C–H bond due to alkanes and aromatic generated two sharp peaks of same intensity at 870 cm^−1^ and 750 cm^−1^. A small band was also pointed at 890 cm^−1^ representing C–H group trisubstituted bonding. The broad peaks from 639 to 500 cm^−1^ are related to Ag-NPs bonding with sulphur from thiol groups of GSH molecules.

### Antioxidant activity

Radical scavenging activity is a measure of scavenging of free radicals which are generated in the form of reactive oxygen species (ROS). DPPH is a free radical capable of accepting an electron or hydrogen radical to become a stable molecule and it has an absorption maximum at 517 nm. Radical Scavenging Activity (RSA) of different synthesized AgNPs viz*.,* clove (green), NaBH_4_ (uncapped) and GSH (capped) were evaluated. The radical scavenging activity was found to be concentration dependent.

**Figure 7:**
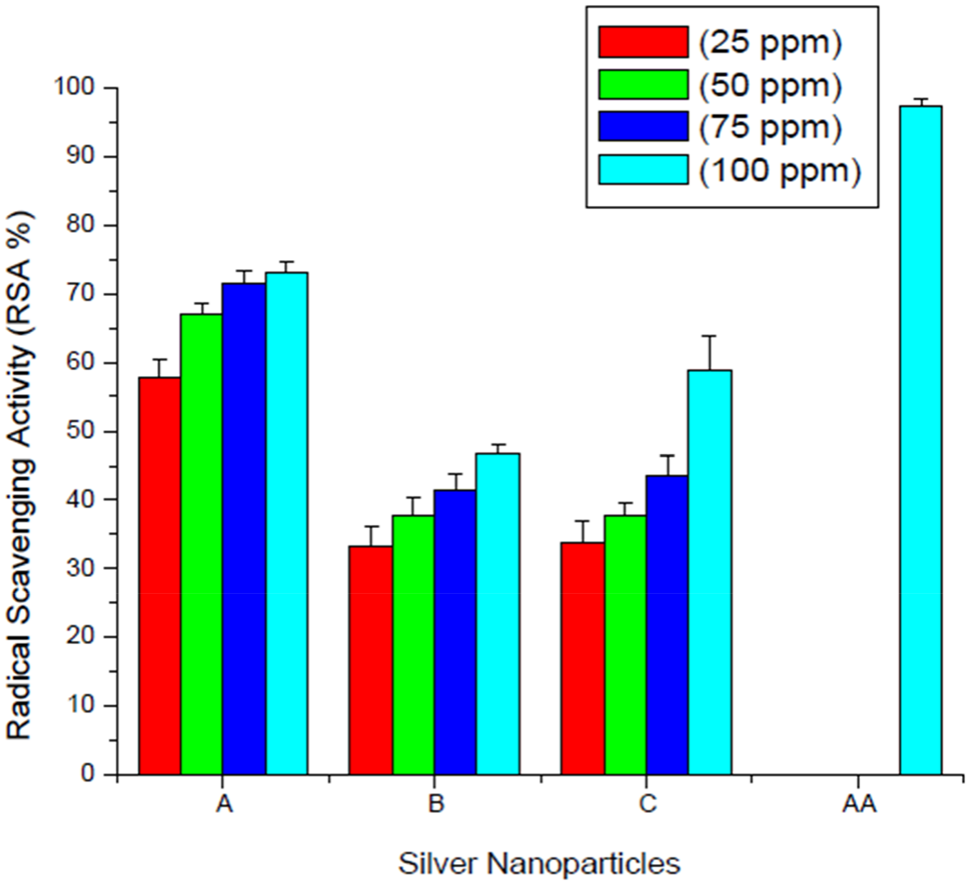
DPPH Antioxidant assay at different concentrations of (A) Clove AgNPs (B) Borohydride AgNPs (C) GSH-capped AgNPs (AA) Ascorbic acid-used as a standard (values are mean ± S.D of three replicates).

Figure 7 depicts the RSA values of the synthesized AgNPs. Ascorbic acid was used as a standard (gave RSA value of 97.17% at 100 ppm conc.) Radical scavenging efficacy of clove AgNPs was found to be the highest among all the samples—58.42% to 74.11%. These results correlate with the other findings done on antioxidant activity of *Leptadenia reticulata* and *Ananas comosus*^25,26^. The percent RSA of chemically synthesized AgNPs i.e., borohydride AgNPs ranged from 33.34 to 46.62%, which was lower than percent RSA value of AgNPs synthesized from clove, moreover the percent RSA values of GSH-capped AgNPs was found to be in the range of 33.89–58.78%, which were lower than the percent RSA values of clove AgNPs but higher than borohydride AgNPs. The decreasing order of radical scavenging activity was as follows: clove AgNPs > GSH-capped AgNPs > Borohydride AgNPs. Chemically synthesized NPs were found to have least percent RSA value (46.62%) among all samples, the reason for this may be accounted to the free ends of chemically synthesized AgNPs which generates free radicals. GSH-capped AgNPs were found to have increased antioxidant efficacy than borohydride AgNPs, which may be due to the capping which probably reduced the free ends of AgNPs resulting in less generation of free radicals. Free radical generation is associated with nanotoxicity and enhancing the antioxidant activity seems to be a promising solution to manipulate the surface properties of nanoparticles imparting stability through thiol rich moieties either chemically or green route.

### Mosquito larvicidal bioactivity

**Figure 8:**
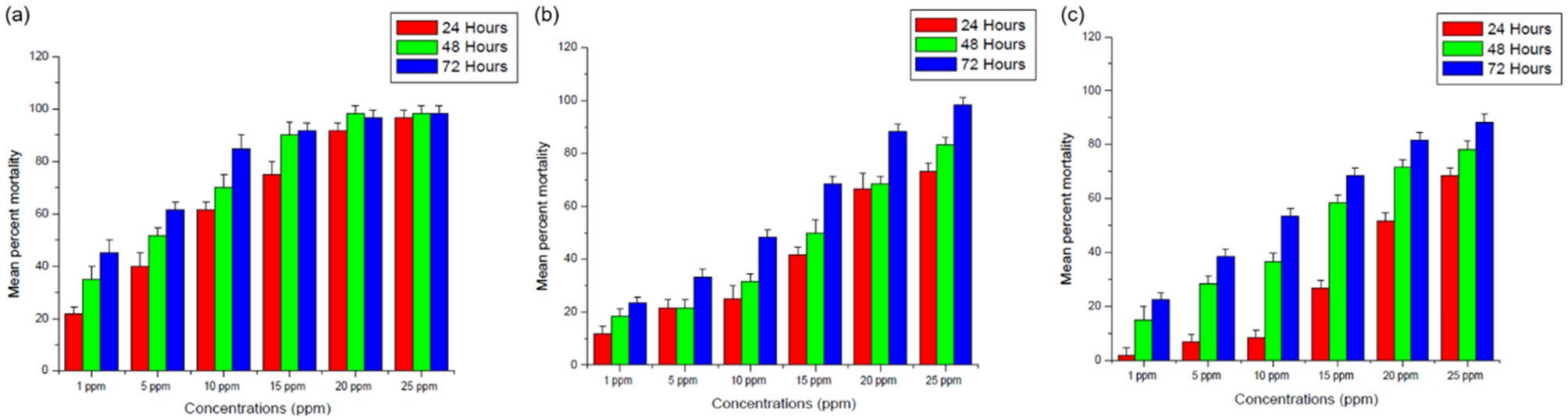
Mean percent mortality at different concentrations at 24 h, 48 h and 72 h of (**a**) Clove AgNPs, (**b**) Borohydride AgNPs, (**c**) GSH-capped AgNPs (values are mean ± S.D of four replicates).

Mosquito larvicidal potential of synthesized AgNPs was determined against III instar larvae of Dengue vector *Aedes aegypti* under laboratory conditions. Control group showed mortality only at 72 h. Mean percent mortality at different concentrations at 24 h, 48 h and 72 h of green, chemically synthesized as well as capped AgNPs was found out. Various mortality patterns were observed at different concentrations and are shown in Fig. 8. Clove mediated AgNPs were found to be very effective and exhibited high mean percent mortality even at 24 h.

At the lowest concentration, clove AgNPs showed mean percent mortality of 21% which reached up to 96% with the highest concentration and at 72 h this range increased from 45 to 98%. Borohydride AgNPs showed lower mean percent mortality as compared to that of clove AgNPs. Their mortality range was found to be between 11 and 73% at 24 h and at 72 h it extended from 23 to 98%. For GSH-capped AgNPs, after 24 h, the mortality was in the range of 1–68%, and then at 72 h it reached from 21 to 88%. Similar results are also reported by various other researchers on dengue vector^27,28^.

Table 1 demonstrates the LC_50_ and LC_90_ values for different AgNPs at different time interval along with lower fiducial limits (LFL) and upper fiducial limits (UFL). Results of the mean percent mortality and LC values clearly indicates that, clove AgNPs being most effective (LC_50_—4.9 ppm, LC_90_—30.2 ppm) followed by GSH-capped (LC_50_—20.13 ppm, LC_90_—46.63 ppm) and borohydride AgNPs (LC_50_—13.43 ppm, LC_90_—160.19 ppm) after 24 h. Green nanoparticles were found more effective owing to the phytopotentials involved. On the other hand, nanoparticles are known to trigger free radical activity on the surface of the particles whereas, capping enhances their scavenging activity as free sites becomes enclosed and reduces the nanotoxicity making them suitable for benign application in various fields. Hitherto, this is the first study where three different types of AgNPs were synthesised using different routes and compared for their different activities and a way is figured out to make the AgNPs much stable and potent for larvicidal applications.

**Table 1:** Larvicidal efficacy (LC_50_ and LC_90_ values) in ppm of various fabricated AgNPs against III instar *Aedes aegypti* larvae at 24 h, 48 h and 72 h.

AgNPs	Hours	LC_50_ (LFL-UFL)	LC_90_ (LFL-UFL)	Equation	Chi-square
Clove AgNPs	24	4.9 (2.88–7.08)	30.2 (18.79–70.28)	3.877x + 1.625	4.25
	48	2.74 (1.26–4.34)	21.14 (13.00–50.30)	4.368x + 1.44	4.93
	72	1.96 (0.672–3.44)	14.69 (8.92–33.47)	4.570x + 1.467	3.71
Borohydride AgNPs	24	13.43 (8.38–25.55)	160.19 (59.72–2618.60)	3.6573x + 1.19	4.87
	48	10.1 (6.09–16.98)	116.22 (48.34–1160.5)	3.786x + 1.21	7.72
	72	3.37 (1.47–5.32)	16.99 (11.16–33.22)	4.038x + 1.823	8.31
GSH-capped AgNPs	24	20.13 (16.69–27.10)	46.63 (32.38–117.76)	0.4188x + 3.513	2.6
	48	9.74 (6.08–15.4)	90.67 (42.21–581.28)	3.692x + 0.756	3.86
	72	6.45 (2.66–10.33)	49.02 (26.53–232.47)	3.823x + 1.45	2.82

*LC*_*50*_* & LC*_*90*_ median lethal concentrations with 50% and 90% mortalities respectively, *LFL* lower fiducial limit, *UFL* upper fiducial limit.

### Acute toxicity of AgNPs to *D. magna*

**Figure 9:**
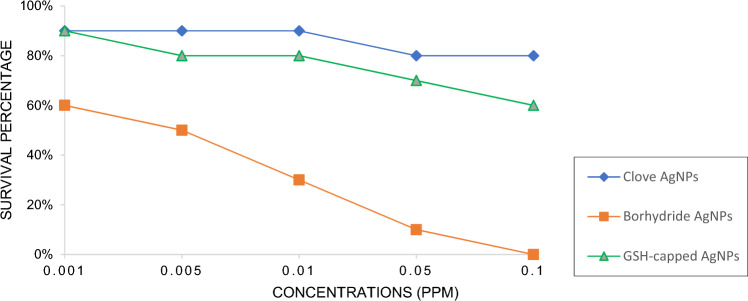
Survival percentage of *Daphnia magna* neonates after 48 h acute toxicity bioassay at different concentrations (ppm) (A) Clove AgNPs, (B) Borohydride AgNPs, (C) GSH-capped AgNPs.

Toxicity assessment of AgNPs is important to understand the risk associated with AgNPs to the environment, hence, 48 h toxicity was evaluated for all the three AgNPs and compared with controls. Percent survivability of *D. magna* neonates treated with various AgNPs, at the highest test concentration depicted 80% and 60% survivability with clove and GSH-capped AgNPs respectively, while, exposure with borohydride AgNPs resulted in 100% mortality. Figure 9 shows the range of percent survivability for all the three AgNPs i.e., clove AgNPs (90–80%), borohydride AgNPs (60–0%) and GSH-capped AgNPs (90–60%) at various test concentrations. Higher survivability percentage with clove AgNPs could be due to the phytopotentials of botanical extract, that posed least threat to the eco-toxicity model organism *D. magna*. On the other hand, higher mortalities on exposure with borohydride AgNPs could be assigned to the chemicals employed during their synthesis, which might have interacted with the cellular and sub-cellular components of *D. magna* and generated oxidative stress, resulting in lesser or no survivability of the test organism. Surprisingly, GSH-capped AgNPs demonstrated higher survivability which was comparable with the green clove AgNPs. It may be deduced from these findings that this moderate to low toxicity could be due to capping of the free ends of these AgNPs, culminating into stabilized AgNPs with least toxicity and safer action as hypothesized in this study. Further, this finds support with the antioxidant activity of GSH-capped AgNPs that was enhanced as compared to the chemically synthesized borohydride AgNPs. The swimming behaviour of *D. magna* neonates was also observed in all three AgNPs treatment groups and compared with that of controls. It was noticed that control and clove AgNPs treated *D. magna* neonates showed normal swimming behaviour both after 24 h and 48 h, while borohydride AgNPs treatment slowed down their movement after 24 h and further after 48 h concentrating mainly at bottom. GSH-capped AgNPs treated neonates showed somewhat transitional swimming behaviour between other two treatment groups.

### Morphological alterations in *D. magna* observed by phase-contrast microscopy

**Figure 10:**
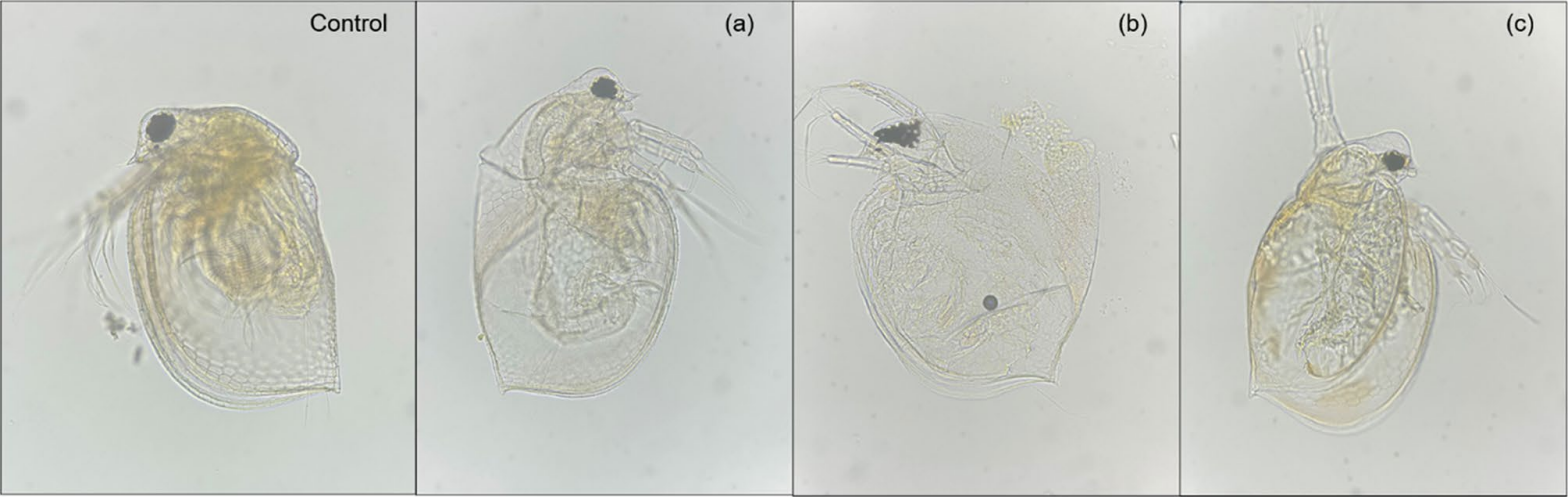
Morphological alterations in *Daphnia magna* neonates after 48 h acute toxicity bioassay observed under phase contrast microscope (**a**) Clove AgNPs, (**b**) Borohydride AgNPs, (**c**) GSH-capped AgNPs.

Morphological alterations of *D. magna* were observed and recorded after 48 h acute toxicity assay (Fig. 10) and compared with that of the control. Control specimen showed healthy neonate with no morphological alterations. *D. magna* following treatment with the clove AgNPs, not much deviation from the controls could be noticed. On the contrary, borohydride AgNPs treated neonates demonstrated various morphological alterations such as overall carapace and lining of internal structures became lighter giving the hyaline appearance as compared to other treatment groups. AgNPs accumulation at the hindgut region, internal fluid leakage from ephippium due to carapace rupturing, disrupted gut region and mouth parts were other major alterations apparently noticeable. Since, *D. magna* is a bottom level filter feeding organism and occupies lower level in food chain and trophic level, any form of nanoparticles accumulation in this organism, may results in transferring nanoparticles and its toxicity to higher level organisms in food chain too^29,30^. Interestingly, GSH-capping to these AgNPs greatly reduced their toxicity as most of the structures were intact and slight pigmentation was found both in foregut and midgut. Major morphological alterations in borohydride AgNPs treated neonates, clearly depicts its higher toxicity as compared to other two AgNPs. The toxicity assay results clearly indicate the non-toxic nature of green AgNPs and also highlights the effectiveness of capping in imparting stability and safety to chemical AgNPs.

## Materials and methods

### Green synthesis of AgNPs

The synthesis of AgNPs was done using a previously reported method, with some modifications^13^. Clove buds were purchased from the local dealer. Various percentages of aqueous extracts (1%, 2%, 3%, 4%, 5% and 10%) were prepared using microwave assisted protocol and filtered. The extract was rich in thiol moieties due to eugenol (4-allyl-2-methoxyphenol) thus acting as a reducing, stabilizing and capping agent and was used for the green synthesis of AgNPs. 10 ml of each of these was mixed with 90 ml of 1 mM silver nitrate (AgNO_3_) solution respectively. The change in colour of solution from colourless to amber and finally to a dark brown colloidal solution indicated the formation of AgNPs. Spectra were taken to monitor the synthesis of extract mediated AgNPs. No distinct absorption peaks could be for 1%, 2%, 3% and 4% however, absorbance and surface plasmon resonance bands were found similar for 5% and 10%, thus 5% was selected for this study taking cost effectiveness into consideration.

### Chemical synthesis of AgNPs

Chemically, AgNPs were prepared using AgNO_3_ as precursor and sodium borohydride (NaBH_4_) as a reducing agent. Briefly, 100 ml of 0.25 mM AgNO_3_ was prepared with continuous stirring. Freshly prepared 0.1 M NaBH_4_ (150 ml) was taken into the conical flask and kept on the ice bath for half an hour. To this solution drop by drop AgNO_3_ was added at the rate of 1 drop/s. Colour change was noticeable (pale brown), which indicated the formation of AgNPs^31^.

### Synthesis of glutathione (GSH)-capped AgNPs

Surface modification of borohydride AgNPs by GSH was done with some modifications in protocol of Chandraker et al.^10^. Briefly, 100 ml AgNO_3_ (0.23 nM) was prepared, to which freshly prepared 10 ml NaBH_4_ (0.1 M) was added, with continuous stirring for 30 min. After that, ethanolic GSH was prepared by mixing 0.05 g of GSH in 10 ml ethanol and it was added to the above solution. pH was adjusted to 11.67, using dilute NaOH. The prepared solution was kept in dark with constant stirring for 20 h. Colour change from pale yellow to dark yellow indicated the AgNPs surface modification by GSH capping.

### Characterization of AgNPs

The nanoparticles were purified by centrifugation and redispersion of pellet in milliQ water. Sample preparation was done for making the sample suitable for different characterizations. UV–Vis Spectrophotometric analysis was done using Systronics double beam spectrophotometer (2203, India) (Department of Zoology, DEI, Agra) for monitoring the reduction of pure silver ions at different intervals. The spectra were measured in wavelength range between 300 and 700 nm. Hydrodynamic size, distribution and stability analysis of AgNPs were evaluated by Zetasizer instrument (Malvern, UK) (SAIF, AIIMS, New Delhi) by measuring DLS and Zeta potential. PdI (Polydispersity Index) was also measured for determining the width of particle size distribution. For DLS analysis samples were diluted with milli Q water (1:4) to avoid multiple scattering effects. Measurements were performed in triplicates. The temperature equilibration time was 1 min at 25 °C and data processing was set to high multi-modal resolution. XRD (X- Ray Diffractometer) measurements were done to study the crystal structure. XRD was performed using a Bruker AXS D8 advance diffractometer, Germany (Central facility Department of Chemistry, DEI, Agra) having Cu Kα 1 (λ = 1.54060 Å) radiation at 40 kV and 30 mA. Detailed morphology and size, shape analysis was done using FE-SEM (Field Emission Scanning Electron Microscope) JEOL JSM-7610FPlus, Japan (Central facility Department of Chemistry, DEI, Agra). Samples were analyzed after drying and platinum coating at 20 kV. Elemental analysis was done using EDXS (Energy Dispersive X-ray Spectroscopy) equipped with FE-SEM. Further TEM (Transmission Electron Microscope) analysis was done to analyze exact shape, size and morphology of different AgNPs. This was done using Thermo Scientific Talos at 200 kV (SAIF, AIIMS, New Delhi) by putting liquid drops solution of the synthesized AgNPs solution on carbon coated copper grids. Copper grids with AgNPs solution were dried and kept under vacuum in desiccator before putting the samples on specimen holder. FTIR (Fourier Transform Infra-red) analysis was also done for finding out presence of various biomolecules and functional groups, responsible for reduction and stabilization in different AgNPs. FTIR analysis was done using Perkin Elmer FT-IR Spectrometer Frontier (AIRF JNU, Delhi).

### Antioxidant activity-DPPH (2,2-di phenyl-1-picrylhydrazyl) assay

Antioxidant activity of AgNPs was determined using the previously reported protocol^10^. Briefly, different concentrations (25–100 ppm) of synthesized AgNPs were prepared and 1.5 ml of these prepared concentrations was added to 1.5 ml of 0.25 mM DPPH solution. Mixtures were incubated in dark for 30 min and colour fading was observed. Absorbance was taken at 517 nm using Bio Spectrometer Eppendorf, Germany (Department of Zoology, DEI, Agra). Ascorbic acid was used as standard. Percent antioxidant activity for each sample was determined using the following formula

$$ {\text{Percent radical scavenging activity}} = \, \left[ {{\text{A}}_{{{\text{DPPH}}}} {-}{\text{ A}}_{{\text{S}}} {\text{/A}}_{{{\text{DPPH}}}} } \right] $$

where, A_S_ = absorbance of DPPH solution with nanoparticles; A_DPPH_ = absorbance of DPPH solution without nanoparticles.

### Laboratory evaluation of mosquito larvicidal bioactivity

Mosquito larvicidal bioactivity was tested for the dengue transmitting mosquito, *Aedes aegypti*. Larvae of *A*. *aegypti* were collected from natural oviposition sites using standard dipping procedure. Third instar larvae were separated by careful examination of their identification key features. Larvicidal bioassay of all three types of AgNPs was carried out following the standard guidelines of World Health Organization^32^. Effectiveness of synthesized AgNPs were tested in 4 batches, 20 larvae (each set), with 200 ml of distilled water for each concentration of different synthesized AgNPs. The experiment was run for 3 days i.e., 24 h, 48 h and 72 h, during this period the rate of percentage mortality was recorded. The percentage mortality was corrected using Abbott’s formula:

$$ \left( {{\text{T}} - {\text{C}}/{1}00 - {\text{C}}} \right) \times {1}00 $$

where, T = Percent mortality in treatment, C = Percent mortality in control.

### *D. magna* acute toxicity test

Acute 48 h toxicity test was done on *D. magna* according to the Organization for Economic Cooperation and Development (OECD, 2004) guideline number 202 (*Daphnia* sp. Acute immobilization act)^33^. Various test concentrations of AgNPs were prepared from stock. A preliminary experiment was set up to decide effective concentration from broad concentration ranges. Following concentrations were selected for the AgNPs toxicity testing—0.001 ppm, 0.005 ppm. 0.01 ppm, 0.05 ppm, 0.1 ppm. Less than 24 h old neonates form third brood progeny were selected. 10 neonates were placed in 250 ml beakers containing 100 ml test concentrations. quadruplicate of each test concentration and control was put. Test were done in optimum conditions temperature (20 ± 2 °C) and 16:8 h light–dark cycles respectively and dissolved oxygen concentration was higher than 3 mg L^−1^ in each test set. No food was given during test period, as organic matters affect intake of NPs in the test organism, thus affecting and wrongly modifying the overall result. Percent survivability was recorded after 24 h and 48 h and swimming behaviours were also observed. Test organism was considered dead, when it showed no observed movement in post-abdomen or appendages within 15 s of gentle agitation of test container^34^. The toxicity tests were considered valid, if mortality in control was less than 10%. Morphological alterations in test organism after 48 h toxicity assay were recorded using OPTIKA phase contrast microscope (IM-3), Italy, having slider with phase Rings (4×/10×, 20×/40×).

### Statistical analysis

Value of mean percent mortality, radical scavenging activity and toxicity assay are represented in mean ± standard deviation. Larvicidal susceptibility is expressed in terms of LC_50_ and LC_90_ using probit analysis^35^. Acute toxicity assay is expressed in terms of survival percentage. Control mortality was corrected using the Abbott’s formula^36^. ORIGIN 9.0 software was used in generating graphs.

## Conclusions

This is first ever comparative study of green and chemically synthesized GSH capped AgNPs for evaluating their bioactivities and eco-toxicity screening. Concludingly, the present study clearly elucidates that among the three types of AgNPs, clove AgNPs had highest antioxidant and mosquito larvicidal bioactivity followed by GSH-capped AgNPs and borohydride AgNPs. This study also highlights that GSH capping greatly reduced toxicity through stabilization of the free ends of chemically synthesized AgNPs validating our hypothesis of safer synthesis of AgNPs. It may be envisaged that utilizing botanicals and also capping of chemical nanoparticles are excellent ways for the synthesis of small sized and effective nanoparticles, and these can be further explored for diverse biomedical and therapeutic applications and bioactivities.

## Data Availability

All data generated or analysed during study are included in this published article however, may be made available on further reasonable request from corresponding author.
